# Usefulness of C-MAC video laryngoscope in direct laryngoscopy training in the emergency department: A propensity score matching analysis

**DOI:** 10.1371/journal.pone.0208077

**Published:** 2018-12-12

**Authors:** Sung Yeon Hwang, Se Uk Lee, Tae Rim Lee, Hee Yoon, Joo Hyun Park, Won Chul Cha, Min Seob Sim, Ik Joon Jo, Keun Jeong Song, Seonwoo Kim, Sun-Young Baek, Tae Gun Shin

**Affiliations:** 1 Department of Emergency Medicine, Samsung Medical Center, Sungkyunkwan University School of Medicine, Seoul, Korea; 2 Department of Emergency Medicine, College of Medicine, Kangwon National University, Chuncheon, Gangwon, Korea; 3 Department of Emergency Medicine, Seoul National University Hospital, Seoul, Korea; 4 Department of Emergency Medicine, Chamjoeun Hospital, Gwangju-si, Gyeonggi-do, Korea; 5 Statistics and Data Center, Samsung Medical Center, Sungkyunkwan University School of Medicine, Seoul, Korea; Imam Abdulrahman Bin Faisal University College of Medicine, SAUDI ARABIA

## Abstract

**Objectives:**

We determined the usefulness of C-MAC video laryngoscope (C-MAC) as a safe training tool for the direct laryngoscopy technique in the emergency department.

**Methods:**

We retrospectively analyzed an institutional airway registry of adult (≥18 years old) patients from April 2014 through October 2016. In this study, the operator used C-MAC as a direct laryngoscope (DL) with limited access to the screen, and the supervisor instructed the operator via verbal feedback while watching the screen. Patients were categorized into the DL group if a conventional DL was used and the C-DL group if a C-MAC used as a DL.

**Results:**

Of 744 endotracheal intubations, 163 propensity score-matched pairs were generated (1-to-n matching: C-DL group, 163 vs. DL group, 428). For the propensity-matched groups, the overall first pass success rate was 69%, while those in the C-DL and DL groups were 79% and 65%, respectively. Overall, multiple attempts were required in 8% of patients, with 4% in the C-DL group and 9% in the DL group. The overall complication rate was 11%, with 4% in the C-DL group and 14% in the DL group. In multivariable analysis, the adjusted odds ratios of C-DL use for first pass success, multiple attempts, and complications were 2.05 (95% confidence interval [CI] 1.18–2.87, p < 0.01), 0.38 (95% CI 0.15–0.94; p < 0.01), and 0.28 (95% CI 0.12–0.63; p < 0.01), respectively.

**Conclusions:**

Our study suggests that the C-MAC could be useful for training residents in the direct laryngoscopy while ensuring patient safety in the emergency department.

## Introduction

Endotracheal intubation (ETI) is an essential method for emergency medicine (EM) physicians to establish definitive airways in critically-ill patients. Historically, the direct laryngoscopy technique has been standard to facilitate ETI [1]. Acquisition of this technique by trainees can be accomplished by on-the-job training under the supervision of an experienced physician. The major limitation of conventional teaching methods is that the supervisor and operator do not share identical views of the anatomy and therefore cannot offer or receive real-time feedback.

Recently, video laryngoscope (VL) has become very popular for airway management [1]. Several studies supporting the use of VL for trainee education have been published [2–4]. One of the most useful benefits of VL for teaching ETI is that the supervisor and operator can share identical views on the monitor, and the supervisor can therefore direct the operator to optimize the glottis view and verify tube placement through the vocal cords.

Although the uses of VL have increased recently in the emergency department (ED), conventional direct laryngoscope (DL) remains the most commonly used device to aid ETI [1]. Despite its importance, the skill is difficult to master, and the incidence of difficult and failed intubations is higher in the emergency setting compared to the operating room [5,6]. EM physicians require structured training to establish competency in DL, but the safety of patients must not be compromised by training. One of the most important indicators of competence, first pass success (FPS), is associated with reduced complications because critically-ill ED patients poorly tolerate prolonged attempts at ETI [7–9]. Efforts should be made to reduce the number of intubation attempts during ETI training.

C-MAC Video Laryngoscope (Karl Storz Endoskope, Tuttlingen, Germany) use the same blades as Macintosh DL, with the video camera located near the distal tip of the blade. The C-MAC can be used either for conventional DL or as a VL [10]. In this study, operators used C-MAC as a DL. The goal of this study was to evaluate the usefulness of C-MAC as a training tool for the direct laryngoscopy in the ED. We hypothesized that this approach would facilitate direct laryngoscopy training while improving patient safety, as indicated by increased FPS rates, fewer instances of multiple attempts, and reduced ETI-related complications, such as esophageal intubation (EI), compared to the conventional training methods [2,11,12].

## Methods

### Study design and setting

This study was approved by the Samsung Medical Center Institutional Review Board (IRB), and the need for informed consent was waived because this was a retrospective study and no interventions were performed (IRB number, 2017-04-051). This study was a single-center, retrospective study performed in an ED from April 1, 2014 to October 30, 2016. The institution was a university-affiliated tertiary teaching hospital located in a metropolitan city with approximately 70,000 ED visits a year. This institution has an accredited 4-year EM residency program. About 400 ETIs are performed per year among adult patients in the ED. Most of the intubations are performed by EM physicians.

In March 2014, a continuous quality-improvement program for emergency airway management was initiated. All ETI cases treated in the ED were registered for data collection and quality improvement activity in our institution’s airway management registry.

### Selection of participants

In this study, ETI cases that met all of the following criteria were included in analysis: 1) patient 18 years of age or older, 2) ETI attempts made by residents, and 3) ETI performed by conventional DL or C-MAC as a DL. Patients who were younger than 18 years old, first intubation attempts performed by an attending EM physician, and intubation methods other than conventional DL and C-MAC as a DL were excluded.

### Methods of measurement

ETI cases were categorized into two groups according to the device used for the first attempt: 1) the DL group when a conventional DL was used; and 2) the C-DL group when a C-MAC was used as a DL with real-time feedback from the supervising physician. A conventional Macintosh blade (size 3 or 4) was used in the DL group, and a Macintosh-type C-MAC blade (size 3 or 4) was used in the C-DL group. D-BLADE (Karl Storz Endoskope, Tuttlingen, Germany) was not used in this study because of its variation from conventional Macintosh blades. The C-MAC blade was connected to the accompanying C-MAC monitor to allow the supervisor to see the operator’s view during ETI. Operators were not allowed to see the monitor and were required to identify the anatomy through the patient’s mouth. The supervisor provided instructions in real time to assist the operator in finding the anatomical landmarks and verifying tube placement through the vocal cords. The supervisor was not allowed to guide the hand of the trainee to assist ETI. If a critical situation was anticipated or occurred during the first attempt, the supervisor stopped the trainee and took over the blade or guided the hand of the trainee, which was recoded as second attempt. In the case of first attempt failure, the operator was allowed access to the screen or was allowed to select another available device.

Stylet is an important issue affecting ETI success rates. Levitan et al. [13] showed that a bending angle greater than 35° with a straight-to-cuff styletted tracheal tube impeded tube passage into the trachea. We standardized the stylet use during the quality improvement program. Malleable steel stylets were used during ETI attempts in all cases in our ED. The tube was usually prepared as a straight-to-cuff shape with bend angles of approximately 30° just proximal to the cuff. The distal end of the stylet was positioned in the middle of the Murphy eye. We stressed the importance of not letting the end of the stylet come out of the tube, which could result in injury the anterior tracheal wall. In the case of difficult ETIs, the operator modified the tube shape or bending angle based on his or her preference.

All ETI procedures in our study were independently monitored by ED medical staff, and the data were collected in real time to minimize recall and reporting bias. Airway management registry was completed at the end of the procedure by the operator and monitoring staff, and the president of the quality improvement program confirmed these data. The following data were retrieved from the airway registry and from electronic medical records by a single abstractor: general characteristics of patients including age, sex, height and weight; indications for intubation; number of intubation attempts; intubating devices; glottic opening score as reported by the operator; presence of difficult airway characteristics; associated complications; operator experience with ETI; sedatives and neuromuscular blocking agents; and level of residency.

Intubation attempts were defined as the placement of a laryngoscope blade into the mouth regardless of successful tube insertion into the trachea. Multiple attempts were defined as three or more intubation attempts. FPS was defined as successful ETI on the first intubation attempt. The term “junior resident” referred to first- and second-year residents, and “senior residents” referred to third- and fourth-year residents. Anticipated difficult airways were defined by the following characteristics: external appearance including short neck, facial trauma, or small mandible; obesity; Mallampati class 3 or higher; airway obstructions including airway edema or a history of tracheal stenosis; distorted airway due to tuberculosis or surgery; cervical immobilization; limited mouth opening less than 3 cm; and lung stiffness including pulmonary edema. Post-intubation hypotension was defined as systolic blood pressure less than 90 mmHg at any time during the 30 minutes following intubation. Post-intubation hypoxemia was defined as peripheral oxygen saturation less than 80% at any time during the 30 minutes following intubation. Cardiac arrest and preexisting hypotension were excluded from definitions of post-intubation hypotension or hypoxemia.

### Outcome measures

The primary outcome measure was FPS rate. The secondary outcome measures were multiple attempts and ETI-related complications.

### Primary data analysis

We present data as mean (standard deviation) for numeric data and number with percentage for categorical data. We used propensity score matching to adjust for patient and operator imbalance between the C-DL group and DL group using variables of indications for intubation, presence of difficult airway characteristics, level of residency (junior vs. senior), and intubation experience of the operator (≤10 times vs. >10 times) [14,15]. We used 1-to-n matching with caliper = 0.2. The weighted generalized estimating equations approach was used to evaluate the relationships between C-DL and primary and secondary outcomes in both univariable analysis and multivariable analysis. For multivariable analysis, we selected covariates that showed differences with p values <0.05 in univariable analysis. P<0.05 was considered to indicate statistical significance. The data were analyzed using SAS version 9.4 (SAS Institute, Cary, NC, USA) and R 3.0.3 (Vienna, Austria; http://www.R-project.org/).

## Results

**Fig 1:**
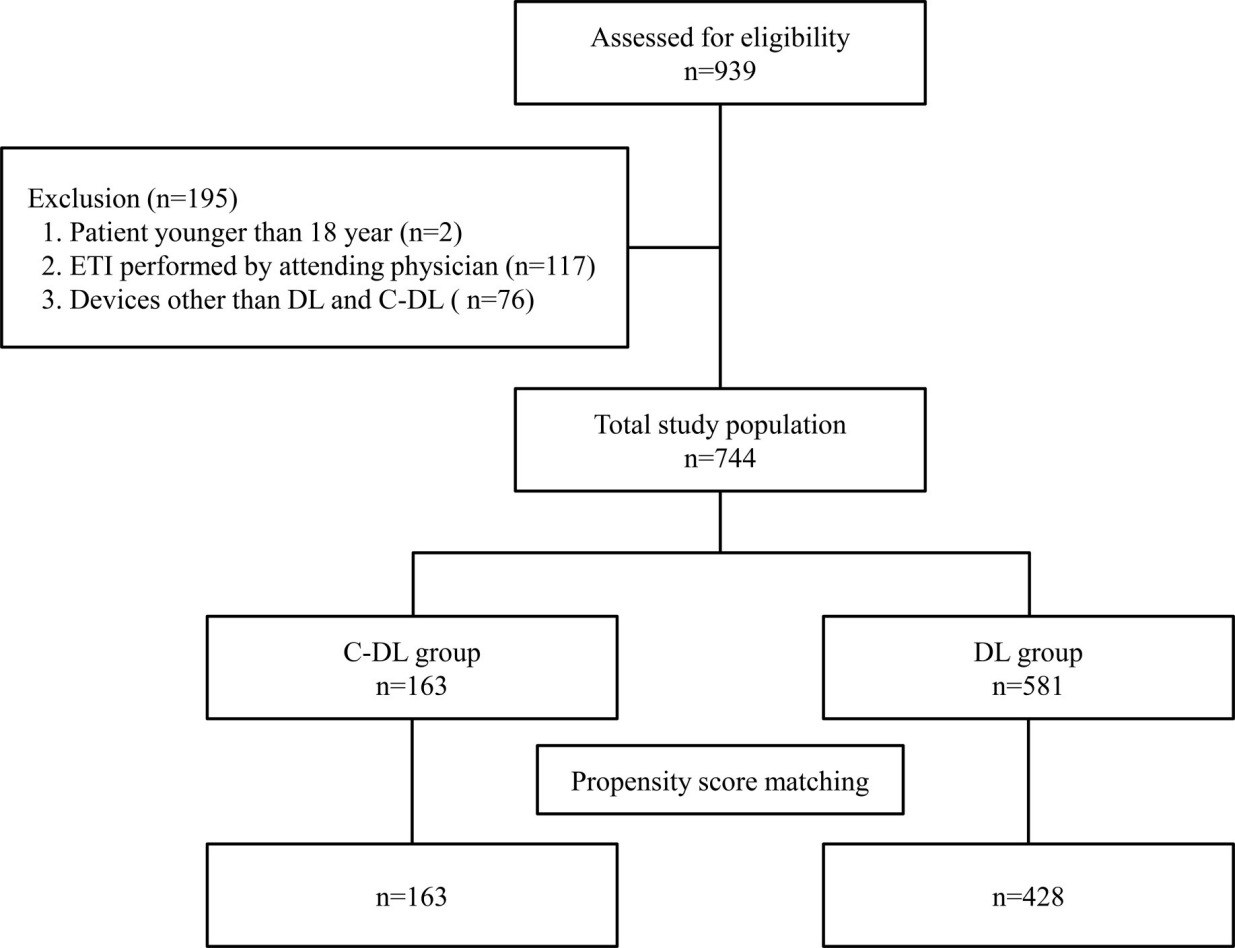
Flow diagram of patients included in the analysis. ETI, endotracheal intubation; DL, conventional direct laryngoscope; C-DL, C-MAC used as a direct laryngoscope.

A total of 939 ETI were performed during the study period. Of these, 195 ETI were excluded, and the remaining 744 ETIs were included in final analysis. Of the eligible patients, 163 used C-DL, and the other 581 patients served as the controls. Among these patients, 163 propensity score-matched pairs were generated (1-to-n matching: C-DL group, 163 vs. DL group, 428) (Fig 1). The baseline characteristics of all patients and matched patients according to propensity score are shown in Table 1. The propensity score matching method resulted in balanced groups for matching variables.

**Table 1:** Baseline characteristics of patients.

	Before matching			After matching*		
	Total (n = 744)	C-DL group (n = 163)	DL group (n = 581)	Total (n = 591)	C-DL group (n = 163)	DL group (n = 428)
**Patient-related factors**						
Age (years)	63 (17)	64 (17)	63 (17)	63 (17)	64 (17)	63 (17)
Sex (male)	462 (62)	103 (63)	359 (62)	376 (64)	103 (63)	273 (64)
Height (cm)	166.0 (12.2)	167.4 (8.8)	165.6 (13.0)	166.0 (12.7)	167.4 (8.8)	165.4 (13.8)
Weight (kg)	62.8 (13.6)	64.7 (13.3)	62.2 (13.7)	62.3 (14.1)	64.7 (13.3)	61.4 (14.3)
POGO score	65 (35)	69 (34)	63 (36)	65 (35)	69 (34)	63 (36)
Intubation indication^†^						
Cardiac arrest	253 (34)	46 (28)	207 (36)	180 (30)	46 (28)	134 (31)
Altered mentation	178 (24)	48 (29)	130 (22)	148 (25)	48 (29)	100 (23)
Respiratory distress	267 (36)	61 (37)	206 (35)	229 (39)	61 (37)	168 (39)
Shock	40 (5)	7 (4)	33 (6)	29 (5)	7 (4)	22 (5)
Other	6 (1)	1 (1)	5 (1)	5 (1)	1 (1)	4 (1)
Anticipated difficult airway^†^	291 (39)	72 (44)	219 (38)	239 (40)	72 (44)	167 (39)
Sedatives						
Ketamine	182 (24)	37 (23)	145 (25)	152 (26)	37 (23)	115 (27)
Etomidate	244 (33)	66 (40)	178 (31)	200 (34)	66 (40)	134 (31)
Midazolam	25 (3)	2 (1)	23 (4)	18 (3)	2 (1)	16 (4)
Other	8 (1)	3 (2)	5 (1)	8 (1)	3 (2)	5 (1)
No sedatives	285 (38)	55 (34)	230 (40)	213 (36)	55 (34)	158 (37)
NMBA						
Succinylcholine	335 (45)	75 (46)	260 (45)	275 (47)	75 (46)	200 (47)
Non-depolarizing NMBA^‡^	124 (17)	38 (23)	86 (15)	105 (18)	38 (23)	67 (16)
No NMBA	285 (38)	50 (31)	235 (40)	211 (36)	50 (31)	161 (38)
**Operator-related factors**						
Intubation experience^†^						
≤ 3	38 (5)	14 (9)	24 (4)	51 (9)	14 (9)	37 (9)
4–10	99 (13)	37 (23)	62 (11)	133 (23)	37 (23)	96 (22)
10–50	287 (36)	88 (54)	199 (34)	274 (46)	88 (54)	186 (44)
> 50	320 (43)	24 (15)	296 (51)	132 (22)	24 (15)	108 (25)
Junior resident^†^^§^	382 (51)	128 (79)	254 (44)	464 (78)	128 (79)	336 (78)

The data are presented as mean (standard deviation) or number (%).

*These are weighted values using a weighted generalized estimating equations approach.

†Propensity score was matched.

‡Rocuronium, vecuronium, and cisatracurium are included.

§ Junior resident refers to first-year and second-year residents.

POGO, percentage of glottic opening; NMBA, neuromuscular blocking agents

### Outcomes

The primary and secondary outcomes are shown in Table 2. Before matching, the overall FPS rate of eligible patients was 72% (n = 539/744). For the propensity-matched groups, the overall FPS rate was 69% (n = 409/591), with 79% (n = 129/163) in the C-DL group and 65% (n = 280/428) in the DL group. Overall multiple attempts occurred in 8% (n = 44/591) of patients, 4% (n = 6/163) in the C-DL group and 9% (n = 38/428) in the DL group. The overall intubation-related complication rate was 11% (n = 65/591), with 4% (n = 7/163) in the C-DL group and 14% (n = 58/428) in the DL group. The immediately-recognized EI rate was only 2% (n = 3/163) in the C-DL group but was 8% (n = 35/428) in the DL group. There were no unrecognized EIs in either group during the study period.

**Table 2:** Outcomes.

	Before matching			After matching		
	Total (n = 744)	C-DL group (n = 163)	DL group (n = 581)	Total (n = 591)	C-DL group (n = 163)	DL group (n = 428)
First pass success rate	539 (72)	129 (79)	410 (71)	409 (69)	129 (79)	280 (65)
Multiple attempts*	57 (8)	6 (4)	51 (9)	44 (8)	6 (4)	38 (9)
Complication	80 (11)	7 (4)	73 (13)	65 (11)	7 (4)	58 (14)
Post-intubation hypotension	13 (2)	1 (1)	12 (2)	9 (1)	1 (1)	8 (2)
Post-intubation hypoxemia	4 (0.5)	1 (1)	3 (0.5)	4 (1)	1 (1)	3 (1)
Vomiting	4 (0.5)	-	4 (1)	5 (1)	-	5 (1)
Esophageal intubation	41 (6)	3 (2)	38 (7)	38 (6)	3 (2)	35 (8)
Agitation	7 (1)	1 (1)	6 (1)	6 (1)	1 (1)	5 (1)
Dental injury	11 (1)	-	11 (2)	9 (1)	-	9 (2)
Cardiac arrest	6 (1)	1 (1)	5 (1)	3 (0.5)	1 (1)	2 (0.5)

The data are presented as number (%).

*Multiple attempts were defined as three or more intubation attempts.

### Associations between C-DL and outcomes

The results of univariable and multivariable analyses for primary and secondary outcomes in the propensity-matched 591 patients are shown in Table 3. The unadjusted odds ratios (OR) of C-DL for FPS rate, multiple attempts, and complications were 2.03 (95% CI 1.28–3.22; p < 0.01), 0.38 (95% CI 0.15–0.93; p = 0.03), and 0.28 (95% CI 0.13–0.63; p <0.01), respectively. In multivariable analysis, the adjusted ORs of C-DL use for FPS rate, multiple attempts, and complication incidence were 2.05(95% CI 1.18–2.87, p < 0.01), 0.38 (95% CI 0.15–0.94; p < 0.01), and 0.28 (95% CI 0.12–0.63; p < 0.01), respectively.

**Table 3:** Relationships between outcomes and C-DL among 591 propensity-matched patients.

	Univariable analysis			Multivariable analysis		
	OR	95% CI	P	OR	95% CI	P
First pass success						
C-DL*	2.03	1.28–3.22	<0.01	2.05	1.18–2.87	<0.01
Patient age	1.00	0.99–1.01	0.62			
Patient sex (female)	1.83	1.18–2.83	<0.01	1.84	1.18–2.87	<0.01
Patient height (cm)	0.99	0.96–1.02	0.59			
Patient weight (kg)	0.99	0.98–1.00	0.07			
Intubation indication			0.24			
Cardiac arrest	Reference					
Altered mental status	1.03	0.64–1.64	0.92			
Respiratory distress	1.56	0.99–2.46	0.05			
Circulatory shock	1.39	0.51–3.79	0.52			
Others	4.12	0.46–36.75	0.20			
Junior resident or novice ^†^	0.66	0.43–1.01	0.06			
Multiple attempts						
C-DL*	0.38	0.15–0.93	0.03	0.38	0.15–0.94	0.04
Patient age	1.00	0.98–1.02	1.00			
Patient sex (female)	0.44	0.21–0.93	0.03	0.44	0.21–0.93	0.03
Patient height (cm)	1.03	0.99–1.06	0.15			
Patient weight (kg)	1.00	0.99–1.02	0.65			
Intubation indication						
Cardiac arrest	Reference					
Altered mental status	0.70	0.30–1.63	0.40			
Respiratory distress	0.62	0.29–1.36	0.24			
Circulatory shock	0.16	0.2–1.37	0.09			
Others	1.06	0.09–11.90	0.96			
Junior resident or novice ^†^	0.87	0.46–1.65	0.67			
Complication						
C-DL*	0.28	0.13–0.63	<0.01	0.28	0.12–0.63	<0.01
Patient age	1.01	1.00–1.03	0.21			
Patient sex (female)	0.48	0.27–0.87	0.02	0.48	0.27–0.86	0.01
Patient height (cm)	1.01	0.99–1.03	0.56			
Patient weight (kg)	1.00	0.99–1.02	0.99			
Junior resident or novice^†^	0.54	0.25–1.14	0.11			

*C-DL, C-MAC used as a direct laryngoscope with real-time feedback by the supervisor.

†Junior resident refers to first-year and second-year residents and novice refers to endotracheal intubation experience of 10 or fewer procedures.

OR, odds ratio; CI, confidence interval

## Discussion

In this study, we evaluated the usefulness of C-MAC in direct laryngoscopy training in the ED. When baseline characteristics including indications of intubation, the presence of difficult airway characteristics, level of residency, and the intubation experience of the operator were adjusted by propensity score, the FPS rate was 79% in the C-DL group and 65% in the DL group. In addition, multiple attempts and complication rates were lower in the C-DL group compared to the DL group. In multivariable analysis, C-MAC used as a DL was associated with increased FPS, fewer instances of multiple attempts, and lower complication rates, both before and after adjustment for additional confounding factors. Therefore, our findings have clinically important implications. Not only can this approach provide direct laryngoscopy training for residents, but it can also improve patient safety, as previous studies have shown that the number of intubation attempts is associated with the incidence of adverse events during emergent ETI [7–9].

Since VL was first introduced as an alternative device for ETI, several studies have demonstrated the advantages of VL over DL. VL use is associated with better glottis exposure, less risk of EI, and a higher FPS rate, particularly in difficult airways [11,16–18]. It can also be used as a rescue method with a high success rate after a failed DL attempt [19,20]. In terms of educational aspects, studies also have proven the usefulness of VL. VL allows all team members in the field to observe the intubation procedure through the screen. In this way, the experienced physician can show the trainee how to intubate, as well as direct the trainee during their intubation attempts. Focusing on VL use as a direct laryngoscopy trainer, however, the studies were limited to operating rooms, and it has not been clearly established in the ED. Howard-Quijano et al. [2] conducted a prospective crossover study including 37 novices to determine whether video-assisted laryngoscopy improved the effectiveness of ETI training in patients under anesthesia. The trainees were not allowed access to the screen, and the intubation procedure was guided by a supervisor’s feedback, including hand positioning and anatomic landmarks. They showed that the success rate of ETI attempts was 69% during video-assisted instruction, with a 55% success rate during non-video-assisted instruction (P = 0.04). EI occurred in 3% of video-assisted intubations and in 17% of conventional intubations (P<0.01). Weiss et al. [12] used a VL prototype with a fiber-optic endoscope in 85 pediatric patients during anesthesia. In that study, the operator intubated under a direct view, and the supervisor instructed the operator while watching the video screen. They suggested that video laryngoscopic monitoring in teaching situations enabled the supervisor to instantly recognize and correct problems related to direct laryngoscopy, tube insertion, and placement and to provide early and precise assistance to the operator according to the monitor findings.

ED is not an optimal environment for providing direct laryngoscopy training while ensuring patient safety compared to the operating theater. First, ETI in the ED is unlikely to be conducted as frequently as it can be during anesthesiology training. Second, this procedure often occurs in unpredictable situations such as cardiac arrest. Third, critically-ill ED patients are at high risk of complications during ETI. ETI is often performed in rapidly deteriorating patients with limited physiologic reserves, which means that these patients do not tolerate delayed or failed intubation. These factors limit the time for instruction and correction during direct laryngoscopy and make it hard for the trainee to become skilled in ETI in the ED. In this study, we focused on the role of C-MAC use as a direct laryngoscopy trainer in the ED. Therefore, our proposed method will provide insight into training residents as well as increasing the safety of patients in the ED. First, the whole sequence of procedures and the psychomotor skills required are identical to those of the conventional technique, which is the most important aspect of C-MAC as a direct laryngoscopy trainer. Second, the supervisor can direct the operator to eliminate uncertainty, resulting in decreased laryngoscopy time and possibly lower complication rates. Finally, the supervisor can confirm tube placement into the trachea or immediately recognize EI [11,12].

One noteworthy advantage of C-MAC is that it is easy to switch from DL to VL [15]. Sakles et al. evaluated the clinical utility of the C-MAC as a DL for ETI compared to a conventional DL [10]. In their study, they stated that the benefit of C-MAC is that it allows quick and easy transition to VL if the DL attempt proves difficult or impossible. Although we did not evaluate this issue in our study, it might be clinically useful even in teaching situations. If the first attempt using C-MAC as a DL is not successful, the operator and supervisor can switch to VL to minimize laryngoscopy time, thereby reducing potential complications.

This study has several limitations that should be considered. First, it was conducted as a single-center study in an academic ED, so the results are not generalizable to other institutions. Second, even though the majority of data were prospectively collected immediately after intubation, some were abstracted after chart review. Third, although we tried to adjust for differences in baseline characteristics using propensity score matching, unmeasured confounding factors might have affected the outcomes. Fourth, we provided a standardized protocol to attending physicians and residents regarding the approach; however, protocols were modified based on the attending physician’s preference and device availability. Attending physician preference was not controlled by the investigator. Also, as our ED had only two C-MAC blades and blade sterilization took one day, there were times when the device was not available. There was, therefore, the potential for selection bias. Fifth, we did not evaluate the resident learning curve for DL, so we were unable to determine whether this method could reduce the number of ETIs required to acquire proficiency. Finally, VL performance can vary according to device characteristics and patient airway conditions. VL showed remarkable differences in efficacy, with some devices having a clinically significant failure rate [21,22]. Therefore, our results should be interpreted with caution with regard to other devices or clinical situations.

## Conclusions

Our study suggests that C-MAC could be useful for training residents in the direct laryngoscopy technique while ensuring patient safety in an ED setting. Our results require further prospective validation due to limitations associated with its retrospective design and use of single-center data.
